# Comparing Endoscopic Sleeve Gastroplasty (ESG) and Laparoscopic Sleeve Gastrectomy (LSG) 30-Day Outcomes and Healthcare Utilization: A Multi-Centered Retrospective Cohort Study of 506,597 Patients

**DOI:** 10.1007/s11695-025-07893-7

**Published:** 2025-04-28

**Authors:** Valentin Mocanu, Emily Jordan, Jerry Dang, Thomas Shin

**Affiliations:** 1 Cleveland Clinic, Cleveland, United States; 2 University of Virginia, Charlottesville, United States

**Keywords:** Endoscopic sleeve gastroplasty, Laparoscopic sleeve gastrectomy, Endobariatrics

## Abstract

**Background:**

While the safety and short-term weight-loss outcomes of endoscopic sleeve gastroplasty (ESG) are now well accepted, the modern uptake and its impact on healthcare utilization continue to remain poorly characterized, particularly in contrast to laparoscopic sleeve gastrectomy (LSG).

**Methods:**

After identifying ESG and LSG cases using a combination of CPT and procedural code variables, non-parsimonious multivariable logistic regression models were conducted to identify predictors of serious complications and outpatient emergency department (ED) visits.

**Results:**

A total of 506,597 patients met inclusion criteria (2285 ESG and 504,312 LSG). ESG patients were younger (42.6 ± 11.8 years versus 45.1 ± 10.7 years, *p* < 0.0001), had a lower BMI (39.5 ± 7.5 kg/m^2^ versus 44.9 ± 7.7 kg/m^2^, *p* < 0.0001), and were primarily female (86.2% versus 81.5%, *p* < 0.0001). Multivariable regression modeling revealed no differences in serious complications between ESG and LSG. ESG was protective against ED visits (OR 0.66; 95% 0.54–0.80; *p* < 0.0001). There were no other differences with respect to mortality or other healthcare metrics, such as outpatient rehydration, between procedures.

**Conclusions:**

Multivariable logistic regression modeling of prospectively collected 30-day outcomes in a large multi-institutional database demonstrates that ESG does not confer additional risk of 30-day serious complications compared to LSG and has lower odds off post-procedural ED utilization in the same comparison.

## Introduction

Endoscopic sleeve gastroplasty (ESG), performed using endoscopic suturing devices, is an FDA-approved endoluminal therapy for patients with obesity, gaining recent popularity due to its safety profile, efficacy, and scalability [1–5]. First described in 2013, ESG involves per-oral plication of the greater curve of the stomach using a variety of suture patterns designed to reduce gastric volume by up to 80% and limit gastric expansion [6, 7]. Yet, while the safety and short-term weight-loss outcomes of ESG are now well accepted [1, 2, 6, 8, 9], the modern uptake and its impact on healthcare utilization continue to remain poorly characterized, particularly in contrast to laparoscopic sleeve gastrectomy (LSG).

Recent societal guidelines published in 2023 by the American Society for Metabolic and Bariatric Surgery (ASMBS) provide a comprehensive evaluation regarding the role of ESG in the face of the ever-growing obesity epidemic [10]. The systematic review of 27 studies demonstrates that ESG may achieve an estimated total body weight loss of up to 20% at 12 months, with concurrent metabolic disease improvement. Serious adverse event rates were less than 3%, with most gastrointestinal side effects resolving within the first week of intervention. Reporting on more nuanced healthcare utilization metrics or trends in adoption, however, were not conducted, with no direct comparison between ESG and other standard-of-care metabolic surgical interventions precluding a true evaluation of healthcare system burden.

To address the current gaps in ESG literature, the aims of the present study were to use the Metabolic and Bariatric Surgery Accreditation and Quality Improvement Program (MBSAQIP) to characterize the modern prevalence, trends, and clinical characteristics of ESG patients versus those receiving laparoscopic sleeve gastrectomy. Secondary objectives were to characterize and evaluate for differences between 30-day outcomes, including emergency department utilization, outpatient intravenous (IV) rehydration therapy, and overall serious complications.

## Methods

### Data Source

Data was extracted from the Metabolic and Bariatric Surgery Accreditation and Quality Improvement Program (MBSAQIP) database from 2020 to 2023 inclusive. The MBSAQIP is the largest clinically international accredited data registry capturing bariatric procedures performed in nearly 1000 North American metabolic surgery centers. Thirty-day data, including pre-, intra-, and post-operative variables, are collected prospectively by trained administrators using variables such as current procedural terminology (CPT) codes, which are uniform international standards used for capturing, reporting, and analyzing procedural data. As part of the ongoing accreditation process, participating centers are subject to scheduled data audits to ensure data integrity and to evaluate for data discrepancies. Patient, surgeon, and center are de-identified, with outcome data only provided at the surgeon and center specific level to address quality initiatives.

### Study Design, Variable Definitions, and Population

All elective primary endoscopic sleeve gastrectomy (ESG) and laparoscopic sleeve gastrectomy (LSG) between 2020 and 2023 were identified in this retrospective cohort study. ESG cases were obtained if the MBSAQIP variables *init_proc_desc* and *surgical_approach* were coded as “Endoscopic gastroplasty” and “Endoscopic” respectively. LSG cases were captured by if the *cpt* variable was listed as 43,775. We then organized our results into two cohorts: ESG and LSG. Patients with open procedures were removed, and only initial primary procedures were included in the study analysis.

A comprehensive list of pre-, intra-, and post-operative factors was extracted and included in the analysis. Pre-operative factors included age, racial status, sex, body mass index (BMI), functional status (independent, partially dependent, and dependent prior to surgery), and the American Society of Anesthesiologists (ASA) physical status classifications, as well as chronic obstructive pulmonary disease (COPD). Metabolic variables included diabetes (non-diabetic and diet-controlled, non-insulin dependent, and insulin-dependent), hypertension, dyslipidemia, gastroesophageal reflux disease (GERD), and obstructive sleep apnea (OSA), as well as a history of venous thromboembolism (VTE). Cardiovascular variables included prior myocardial infarction (MI), prior cardiac surgery, and percutaneous coronary intervention (PCI). Other variables, including oxygen dependence, chronic steroid use, anticoagulation status, prior cardiac surgery, renal insufficiency, acute kidney injury (AKI), prior thromboembolic disease, and dialysis dependence, were also included. Intra-operative factors included operative time.

Thirty-day complications included in the study were leak, bleed, cardiac complications, pneumonia, acute kidney injury (AKI), presence of emergency room visits, outpatient dehydration treatment, myocardial infarction, renal failure, pulmonary embolism, wound disruption, surgical site infections (SSI), urinary tract infections, venous thromboembolism, and sepsis. Additionally, 30-day reoperation, intervention, readmission, mortality rates, and a composite variable of overall serious complications were assessed.

### Statistical Analysis

Categorical variables were expressed as absolute values and percentages, and univariate analysis was conducted using chi-squared tests. Continuous variables were expressed as weighted means and standard deviations (SD), and analysis was performed using independent two-sample *t*-tests.

A non-parsimonious multivariable logistic regression model was developed using a hypothesis-driven purposeful selection methodology to evaluate differences in 30-day serious complications between ESG and LSG. Univariate analysis of variables with a *p*-value < 0.10 or from variables previously deemed clinically relevant to our primary outcome was used to generate a preliminary main effects model. Significant variables in the multivariable model were then identified (Wald test *p* < 0.05), and the linear assumption of continuous variables and multi-collinearity were checked using the variance inflation factor (VIF). Variables with a VIF greater than 10 were further explored using collinearity diagnostic tests and excluded from the final model if deemed collinear. The Brier score and the receiver operating characteristic (ROC) curve were used to assess goodness of fit. All statistical analysis was performed using Stata 17 (STATACorp LP, College Station, TX, USA).

### Ethical Approval and Informed Consent

As this was a retrospective study, a formal consent is not required. All procedures performed that contributed to the data registry were in accordance with the ethical standards of the institutional and/or national research committee and with the 1964 Helsinki declaration and its later amendments or comparable ethical standards. This investigation was exempt from Institutional Review Board approval due to the anonymity of deidentified data.

## Results

### Clinical Characteristics and Univariate Analysis of ESG and LSG Cohorts

A total of 506,597 patients met inclusion criteria, with 2285 patients receiving ESG and 504,312 receiving LSG (Table 1). ESG patients were younger (42.6 ± 11.8 years ESG vs. 45.1 ± 10.7 years LSG *p* < 0.0001), had a lower BMI (39.5 ± 7.5 kg/m^2^ ESG vs. 44.9 ± 7.7 kg/m^2^ LSG; *p* < 0.0001), and had a female predominance (86.2% ESG vs. 81.5% LSG; *p* < 0.0001). A smaller proportion of White (54.5% ESG vs. 61.6% LSG; *p* < 0.0001) and Black (16.3% ESG vs. 21.8% LSG; *p* < 0.0001) racial status patients received ESG. Patients who underwent ESG also had an overall reduced metabolic disease burden, with a reduced prevalence of sleep apnea (17.6% ESG vs. 35.0% LSG; *p* < 0.0001), insulin and medication-dependent diabetes (12.4% ESG vs. 20.6% LSG; *p* < 0.0001), hypertension (30.8% ESG vs. 42.0% LSG; *p* < 0.0001), and dyslipidemia (14.4% ESG vs. 20.9% LSG; *p* < 0.0001).

**Table 1:** Clinical characteristics of ESG and LSG cohorts

	LSG (*n* = 504,312)	ESG (*n* = 2285)	
			*p*-value
Age (mean (SD))	42.6 (11.8)	45.1 (10.7)	< 0.0001
< 18	1549 (0.3)	2 (0.1)	< 0.0001
18–30	66,953 (13.3)	151 (6.6)	
30–40	148,906 (29.5)	570 (25.0)	
40–50	141,932 (28.1)	797 (34.9)	
50–60	98,602 (19.6)	531 (23.2)	
> 60	46,379 (9.2)	234 (10.2)	
Female	411,475 (81.6)	1970 (86.2)	< 0.0001
Racial status			< 0.0001
White	310,430 (61.6)	1245 (54.5)	
Black	110,111 (921.8)	372 (16.3)	< 0.0001
Other	83,771 (16.6)	668 (29.2)	
BMI (mean (SD))	44.9 (7.7)	39.6 (7.5)	< 0.0001
< 35	20,414 (4.1)	730 (32.0)	
35–40	119,361 (23.7)	653 (28.9)	
40–50	158,030 (31.3)	425 (18.6)	
50–60	100,946 (20.0)	251 (11.0)	< 0.0001
60–70	81,910 (16.2)	199 (8.7)	
> 70	23,644 (4.7)	27 (1.2)	
Functional status			0.1
Independent	501,465 (99.5)	2279 (99.8)	
Partially dependent	2296 (0.5)	4 (0.2)	
Dependent	113 (0.02)	0 (0)	
ASA category			< 0.0001
ASA 1–2	99,166 (19.7)	1005 (44.4)	
ASA 3	387,592 (77.0)	1135 (50.2)	
ASA 4–5	16,764 (3.3)	123 (5.4)	
Diabetes			
Non-diabetic and diet-controlled	400,324 (79.4)	2000 (87.5)	< 0.0001
Non-insulin-dependent	79,267 (15.7)	220 (9.6)	
Insulin-dependent	24,721 (4.9)	65 (2.8)	
HTN	211,916 (42.0)	704 (30.8)	< 0.0001
GERD	126,645 (25.1)	627 (27.4)	0.01
COPD	5273 (1.1)	7 (0.3)	< 0.0001
DLD	105,411 (20.9)	329 (14.4)	< 0.0001
Renal insufficiency			0.001
Dialysis	1811 (0.4)	4 (0.2)	0.1
Prior VTE	12,122 (2.4)	41 (1.8)	0.06
Therapeutic anticoagulation	14,064 (2.8)	35 (1.5)	< 0.0001
Sleep apnea	176,661 (35.0)	403 (17.6)	< 0.0001
Prior MI	4682 (0.9)	15 (0.7)	< 0.0001
Prior cardiac surgery	3905 (0.8)	12 (0.5)	0.2
Operative year			0.02
2020	103,746 (20.6)	425 (18.6)	
2021	129,862 (25.8)	602 (26.4)	
2022	140,493 (27.9)	691 (30.2)	
2023	130,111 (25.8)	567 (24.8)	
Operative length (hr, mean (SD))	69.3 (36.0)	81.2 (48.2)	< 0.0001
Day of discharge from procedure (mean (SD))	1.21 (0.97)	0.19 (0.92)	< 0.0001

*ASA* American Society of Anesthesiologists, *BMI* body mass index, *COPD* chronic obstructive pulmonary disease, *DLD* dyslipidemia, *ESG* endoscopic sleeve gastroplasty, *GERD* gastroesophageal reflux disease, *HTN* hypertension, *LSG* laparoscopic sleeve gastrectomy, *MI* myocardial infarction, *PCI* percutaneous coronary intervention, *VTE* venous thromboembolism

The delivery of both procedures increased from 2020 to 2022 until a peak of 141,284 cases in the 2022 year, with a total of 691 ESGs performed. Both LSG (*n* = 130, 111, 25.8%) and ESG (*n* = 567, 24.8%) case volumes dropped in the following 2023 operative year. An operative length for ESG cases was significantly longer than LSG (81.2 ± 48.3 min ESG vs. 69.3 ± 36.0 min LSG *p* < 0.0001). Days in hospital following the procedure were significantly longer for the LSG cohort (1.21 ± 0.97 days LSG vs. 0.19 ± 0.92 days ESG; *p* < 0.0001).

### Bi-variate Analysis of Post-Operative Complications in ESG and LSG Cohorts

Differences in 30-day outcomes between ESG and LSG are summarized in Table 2. No significant differences in rates of leak, bleed, cardiac complications, pneumonia, AKI, acute renal failure, pulmonary embolism, urinary tract infections, myocardial infarctions, sepsis, venous thromboembolism, outpatient dehydration treatments, mortality, or serious complications were found between procedures. Similarly, no differences were observed between procedures with regard to 30-day re-interventions or reoperations. LSG was associated with increased rates of superficial SSIs (0.25% LSG vs. 0% ESG; *p* = 0.02) and emergency room outpatient visits (7.2% LSG vs. 4.6% ESG; *p* < 0.0001). In contrast, ESG was associated with increased readmission rates at 30 days (2.9% ESG vs. 2.2% LSG; *p* = 0.02).

**Table 2:** Thirty-day rates of complications for ESG and LSG cohorts

	LSG (*n* = 504,312)	ESG (*n* = 2285)	
			***p*-value**
Complication			
Leak	878 (0.2)	4 (0.2)	1
Bleed	3294 (0.7)	13 (0.6)	0.6
Reoperation	3023 (0.6)	15 (0.7)	0.7
Reintervention	2,201 (0.4)	16 (0.7)	0.06
Readmission	11,114 (2.2)	67 (2.9)	0.02
Emergency visits	36,457 (7.2)	104 (4.6)	< 0.0001
Outpatient dehydration treatments	18,036 (3.6)	82 (3.6)	1
Myocardial infarction	135 (0.03)	0 (0)	0.4
Acute renal failure	180 (0.04)	0 (0)	0.4
Pulmonary embolism	489 (0.1)	5 (0.2)	0.06
Pneumonia	509 (0.1)	3 (0.1)	0.7
Wound disruption	168 (0.03)	0 (0)	0.4
Sepsis	348 (0.1)	2 (0.1)	0.7
Unplanned intubation	310 (0.1)	2 (0.1)	0.6
Venous thromboembolism	1865 (0.4)	12 (0.5)	0.2
Serious complication	10,127 (2.0)	52 (2.3)	0.4
Death	282 (0.1)	1 (0.04)	0.8

### Multivariable Logistic Regression Analysis for Predictors of Serious Complications

Multivariable logistic regression analysis (Table 3) revealed the following top five independent predictors of 30-day serious complications: renal insufficiency (OR 2.29; 95% CI 1.96–2.66; *p* < 0.0001), history of VTE (OR 1.87; 95% CI 2.05–1.71; *p* < 0.0001), partially dependent functional status (OR 1.84; 95% CI 1.53–2.21; *p* < 0.0001), prior MI (OR 1.69; 95% 1.47–1.94; *p* < 0.0001), and COPD (OR 1.60; 95% CI 1.40–1.82; *p* < 0.0001). Female sex (OR 0.88; 95% CI 0.84–0.93; *p* < 0.0001) and non-White/non-African American racial status (OR 0.91; 95% CI 0.86–0.97; *p* < 0.0001) were the only protective factors, while neither ESG nor LSG was significantly associated with serious complications after adjusting for comorbidities. The receiver operating characteristic and Brier score for the model were 0.61 and 0.02, respectively.

**Table 3:** Multivariable logistic regression model for serious complications

Predictors of serious complications	Odds ratio	95% confidence interval	*p*-value
Older age (per 10 years)	1.1	1.08–1.12	< 0.0001
Higher BMI (per 5 kg/m^2^)	1.02	1.00–10.3	0.007
ESG vs. LSG	1.2	0.89–1.56	0.07
Female	0.88	0.84–0.93	< 0.0001
Race			
Black vs. White	1.24	1.18–1.30	< 0.0001
Other vs. White	0.92	0.86–0.97	0.004
Diabetes			
Non-insulin-dependent vs. non-diabetic/diet-controlled	1.05	1.00–1.11	0.07
Insulin-dependent vs. non-diabetic/diet-controlled	211.32	1.22–1.42	< 0.0001
Prior MI	1.69	1.48–1.94	< 0.0001
HTN	1.23	1.17–1.28	< 0.0001
Functional status			< 0.0001
Partially dependent vs. independent	1.84	1.53–2.21	< 0.0001
Dependent vs. independent	1.6	1.71–2.05	0.3
COPD	1.6	1.40–1.82	< 0.0001
Renal insufficiency	2.23	1.96–2.66	< 0.0001

*BMI* body mass index, *COPD* chronic obstructive pulmonary disease, *ESG* endoscopic sleeve gastroplasty, *HTN* hypertension, *DLD* dyslipidemia, *LSG* laparoscopic sleeve gastrectomy, *MI* myocardial infarction

### Multivariable Logistic Regression Analysis for Predictors of Emergency Visits

After adjusting for comorbidities and complications, the five greatest significant contributors of emergency visits were the presence of serious complications (OR 4.70; 95% CI 4.49–4.93; *p* < 0.0001), female sex (OR 1.52; 95% 1.47–1.57; *p* < 0.0001), COPD (OR 1.42; 95% CI 1.28–1.57; *p* < 0.0001), Black racial status (OR 1.31; 95% CI 1.27–1.34; p < 0.0001), and insulin-dependent diabetes (OR 1.27; 95% 1.21–1.33; *p* < 0.0001) (Table 4). Only two protective factors, including ESG (OR 0.66; 95% 0.54–0.80; *p* < 0.0001) and older age (OR 0.81, 95% CI 0.80–0.82; *p* < 0.0001) were identified. The ROC of the model was 0.999, and the Brier score was 0.016, demonstrating excellent model performance.

**Table 4:** Multivariable logistic regression model for emergency visits

Predictors of emergency visits	Odds ratio	95% confidence interval	*p*-value
Older age (per 10 years)	0.81	0.80–0.82	< 0.0001
Higher BMI (per 5 kg/m^2^)	1.02	1.02–1.03	< 0.0001
ESG vs. LSG	0.66	0.54–0.80	< 0.0001
Female	1.52	1.47–1.57	< 0.0001
Race			
Black vs White	1.31	1.27–1.34	< 0.0001
Other vs. White	1.05	1.02–1.08	0.001
Diabetes			
Non-insulin dependent vs. non-diabetic/diet-controlled	1.04	1.01–1.08	0.008
Insulin-dependent vs. non-diabetic/diet-controlled	1.27	1.21–1.33	< 0.0001
Prior MI	1.27	1.14–1.42	< 0.0001
HTN	1.11	1.08–1.14	< 0.0001
Functional status			< 0.0001
Partially dependent vs. independent	1.24	1.07–1.43	0.004
Dependent vs. independent	1.23	0.65–2.33	0.5
COPD	1.42	1.28–1.57	< 0.0001
Renal insufficiency	1.26	1.10–1.44	0.001
Serious complications	4.7	4.49–4.93	< 0.0001

*BMI* body mass index, *COPD* chronic obstructive pulmonary disease, *ESG* endoscopic sleeve gastroplasty, *HTN* hypertension, *DLD* dyslipidemia, *LSG* laparoscopic sleeve gastrectomy, *MI* myocardial infarction

## Discussion

The present study utilizes a large multi-institutional database of prospectively collected 30-day outcomes to characterize current trends in ESG at metabolic and bariatric surgery centers across North America, in addition to a direct comparison of the modalities’ effect modification on 30-day complications and ED utilizations. The novelty of this study rests in utilizing MBSAQIP data collected inclusive of 2020 to 2023, during which time higher levels of data granularity allow for tracking of endoscopic bariatric procedures.

While there were no significant differences in 30-day reinterventions and reoperations between ESG and LSG, the latter procedure was associated with higher ED visits and SSI. Of note, ESG was associated with increased rates of 30-day readmission, but its significance likely remains only statistical rather than clinical, given the very small difference in readmissions between the two groups. Multivariable logistic regression redemonstrates well-accepted patient factors that are canonically indicative of 30-day complications, including renal insufficiency, VTE, prior MI, COPD, and non-independent functional status. More interestingly, the same analysis reveals ESG is not a significant predictor of 30-day complications despite its longer procedural time and relative novelty compared to LSG. In fact, proceeding with an ESG is a significant protective factor against 30-day ED utilization in multivariable comparison to LSG.

Complications and ED visits are well-known principal contributors to increased healthcare utilization in postoperative patients, amounting in some studies to upwards of $65,000 in total 6-month risk-adjusted healthcare payments related to post-bariatric surgery admissions [11–15]. While previous studies have examined associations between ESG and perioperative outcomes, this present study examines ESG and LSG in direct comparison across numerous centers over a 4-year period [16, 17]. As an outpatient bariatric procedure, ESG has garnered much recent interest, particularly with the rise of novel medical and non-surgical interventions and efforts in maximizing healthcare resource efficiency. While cost data is not directly available in MBSAQIP, this study presents novel findings from the largest multi-institutional database that ESG does not posit increased 30-day postoperative complications or ED visits compared to LSG—both major contributors to postoperative costs. The multivariable logistic models used demonstrate robust goodness-of-fit and identify other well-accepted clinical predictors of complications and ED utilization, lending support and validation to our results [18–20].

It is important to interpret the above findings in context of the patients included in this study. While ESG and LSG case volumes dropped slightly in 2023, the procedures’ patient populations appear disparate. ESG patients were statistically younger and comprised of more females compared to LSG. Perhaps more clinically relevant is the statistically lower BMI and patient cohorts of Black race, obstructive sleep apnea, insulin-dependent diabetes, hypertension, and hyperlipidemia. Overall, these factors are correlative with higher severity of metabolic syndrome and disadvantageous social determinants of health, suggesting ESG cohort may inherently be a healthier cohort thus driving differences in perioperative outcomes [21–24]. These differences may also be a consequence of various provider and financial factors leading to inequitable access to ESG. Future investigations utilizing payor status and other social determinants of health would be helpful in exploring how inequity may play a role in these results.

There are additional limitations to this present study that must be considered, largely inherent to its utilization of a retrospective review of a prospectively collected database. While several statistically significant factors were identified, the rationale for their contributions could not be evaluated within the study design of this work, and future studies are needed in order to better understand their contribution. Reliance on a retrospective method severely limits the study’s ability to account for cohort disparities, especially those of large clinical relevance, such as the severity of metabolic disease and BMI. While the inclusion of 2023 data provides the highest data granularity possible in MBSAQIP, the study is limited to covariates included in the 2020–2023 dataset, with a limited perspective of 30-day outcomes. With the absence of longer term weight and metabolic outcomes, it is difficult to make any definitive conclusions on the clinical or cost equipoise of ESG compared to LSG. Indeed, further data on mid- and long-term ESG complications, weight loss, and amelioration of comorbidities are necessary to allow for a more robust comparison between procedures. Additionally, the study findings are limited to ESG cases performed at participating MBSAQIP bariatric centers and exclude ESG performed by bariatric endoscopists and gastroenterologists at non-participating centers, thus not inclusive of the entire ESG experience at large. However, the use of MBSAQIP has allowed for one of the largest comparisons to date between ESG and LSG in terms of perioperative outcomes and safety, furthering current understanding and discussion of the two procedures’ comparability within a larger algorithm of bariatric and metabolic interventions.

Retrospective review of prospectively collected 30-day outcomes in a large multi-institutional database inclusive of bariatric centers across North America over 4 years provides novel insight into the safety and efficacy of ESG compared to LSG. Multivariable logistic regression modeling demonstrates that ESG does not confer additional risk of 30-day serious complications compared to LSG, and in fact, may be protective against post-procedural ED utilization in the same comparison.

## Conclusion

There are no significant differences in rates of 30-day serious complications between endoscopic sleeve gastroplasty and sleeve gastrectomy; although, gastroplasty was associated with decreased odds of emergency department utilization. These findings highlight the need for further prospective investigations comparing the two procedures to better guide bariatric interventions selection to optimize perioperative outcomes, safety, and healthcare costs.

## Data Availability

The MBSAQIP data within the manuscript is accessible to all accredited centers.
